# Fertilization Strategies in Huanglongbing-Infected *Citrus latifolia* and Their Physiological and Hormonal Effects

**DOI:** 10.3390/plants14071086

**Published:** 2025-04-01

**Authors:** Luis A. Pérez-Zarate, Aída Martínez-Hernández, Francisco Osorio-Acosta, Eliseo García-Pérez, Fredy Morales-Trejo, Juan A. Villanueva-Jiménez

**Affiliations:** 1 Colegio de Postgraduados, Campus Veracruz, Km. 88.5 Carretera Fed. Xalapa-Veracruz, Manlio F. Altamirano, Veracruz 91690, Mexico; alfredo.perez@colpos.mx (L.A.P.-Z.); fosorioa@colpos.mx (F.O.-A.); geliseo@colpos.mx (E.G.-P.); fredymt@hotmail.com (F.M.-T.); 2 Colegio de Postgraduados, Campus Campeche, Carretera Haltún-Edzná Km 17.5, Sihochac, Champotón, Campeche 24450, Mexico; aida.martinez@colpos.mx

**Keywords:** *Candidatus* Liberibacter asiaticus, Persian lime, phytohormones, macronutrients, micronutrients

## Abstract

Huanglongbing disease (HLB), caused by *Candidatus* Liberibacter asiaticus (*C*Las), affects all commercial citrus species. Persian lime (*Citrus latifolia* Tanaka), a crop of global economic importance, has shown tolerance to this disease. Efforts are focused on extending the productive life of diseased trees through effective agronomic management. This study aimed to evaluate how different fertilization strategies influence the physiological and hormonal responses of *Citrus latifolia* on both healthy and HLB-affected plants. It compared the effects of low (Ma-1), medium (Ma-2), and high (Ma-3) doses of macronutrients, with and without the addition of micronutrients (Mi-1), using either soil (Mi-2) or foliar (Mi-3) applications. Treatments were applied every 18 days for one year. *C. latifolia* showed tolerance; however, HLB infection negatively affected growth parameters, photosynthetic activity, vascular bundle anatomy, reflectance at 550 and 790 nm, carbohydrate metabolism, and the concentration of salicylic acid and its biosynthetic precursors. The hormonal response showed higher levels of benzoic acid and lower levels of salicylic acid than those reported in susceptible citrus. Plants treated with low doses of macronutrients along with soil-applied micronutrients (Ma-1 + Mi-2) showed a 17.9% increase in growth, a 31.3% larger canopy volume, and an 83.3% reduction in starch accumulation compared to the treatment with high doses of macronutrients and both soil and foliar applied micronutrients (Ma-3 + Mi-3). These findings indicate that split soil fertilization with low-dose macronutrients and micronutrients might influence plant physiological responses, potentially improving disease management and decreasing fertilizer inputs.

## 1. Introduction

Huanglongbing (HLB) is one of the most devastating diseases in citrus production, causing severe yield losses and substantial economic damage [1]. This disease is caused by a Gram-negative *α*-proteobacteria that is restricted to the phloem [2]. *Candidatus* Liberibacter asiaticus (*C*Las) is the species associated with HLB in Mexico and is transmitted by *Diaphorina citri* Kuwayama, the Asian citrus psyllid [3]. Citrus production profitability has declined due to the high costs of managing the disease in affected areas [4]. HLB is now present in nearly all citrus-producing countries; however, in most regions, the disease remains confined to specific areas [5]. It has resulted in substantial economic losses for countries like China, Brazil, India, and Mexico [6]. In the latter, HLB is present in all 24 citrus-producing states [6].

HLB-infected plants exhibit physiological impairments that lead to foliar symptoms, including green islands, blotchy mottle, lightening and thickening of veins, death of branches and secondary roots, deformation, uneven ripening, and premature fruit drop [1,7]. These symptoms, caused by phloem blockage and restricted photoassimilate flow, disrupt carbohydrate metabolism. Excessive starch accumulation in leaves leads to chloroplast degradation and loss of chlorophyll [8]. Photoassimilate flow to the roots decreases, affecting their development with limited water and nutrient absorption. This limitation may arise from the thickening of xylem cell walls, narrowing the vessel’s lumen, limiting the flow of water and nutrients, and ultimately causing water deficits [9].

HLB symptoms are different between HLB-tolerant and -susceptible citrus. Reports indicate that tolerant varieties could have a specific recognition system for *C*Las and a more efficient detoxification system. This system reduces most damage caused by reactive oxygen species (ROS) and promotes the induction of the immune system, leading to a lower expression of foliar symptoms [10]. Furthermore, tolerant varieties could suppress the direct defense response to *C*Las infection, especially salicylic acid-mediated signaling [11]. However, no *C*Las effectors directly related to foliar symptoms have been identified, which has led to the hypothesis that HLB may act as an autoimmune disease [12].

In tolerant varieties, such as Persian lime, symptom development is slower, and diseased plants maintain their development and production despite suffering from the disease [13]. Furthermore, fewer effects of HLB on Persian lime are present at the subcellular level than in susceptible varieties such as Mexican lime [14]. Despite the above, Persian lime plants infected with HLB reduce fruit yield by approximately 2.4 t ha^−1^ due to a 17.3% reduction in fruit weight and 18.6% in juice volume, a reduction that has been similar in the states of Yucatan and Veracruz [5,15]. In Mexico, proposed HLB management included (a) monitoring and controlling the insect vector, (b) using plants from a nursery certified to mass produce disease-free plants, and (c) promptly removing diseased plants in the early stages of infection. To promote coexistence with HLB, cultural practices were implemented, such as improved fertilization strategies, all within the framework of the regulatory campaign against regulated citrus pests. This was further supported by the establishment of Phytosanitary Epidemiological Management Areas (AMEFIs, its acronym in Spanish) [6,16].

Balanced nutrition is crucial for maintaining plant health and vigor; healthy plants are known to be less susceptible to pathogen attacks [17]. Mineral nutrients are part of the plant’s first line of defense against pathogens since they are required to properly activate defense mechanisms such as producing toxins, metabolites, and lignin. As an example, calcium serves as a key structural component of the cell wall, critical for its integrity and function, while zinc, iron, and manganese act as cofactors in the synthesis of secondary metabolites [18]. Therefore, balanced fertilization has been suggested as a strategy to extend the production of trees affected by HLB. Mineral nutrition can be supplied through granular fertilizers added to the soil, as well as via the foliar application of macro- and micronutrients [19]. It is relevant to note that balanced nutrition is a complementary tool in the HLB management strategy. It works alongside other approaches, such as certified plant material use and biological and chemical vector control [3].

HLB-infected plants show a reduced availability of micronutrients in symptomatic leaves, including Ca^2+^, Mg^2+^, Fe^2+^, Zn^2+^, and Cu^2+^ [20]. Applying nitrogen, phosphorus, potassium, calcium, magnesium, copper, iron, and zinc to HLB-infected trees might mitigate the severity of the disease and enhance fruit production. This improvement might result from fewer disruptions in carbohydrate metabolism and a reduced impact of the disease on plant growth and yield [21,22,23,24]. The foliar and soil application of calcium, magnesium, zinc, and boron also significantly increased root lifespan [25]. High doses of Mn (4X) were associated with reduced *C*Las titers [26]. Thus, a 45.0% increment in fruit production in diseased plants was achieved with micronutrient foliar applications. However, applying doses that exceed recommended levels might reduce fruit yield by up to 25.0% [27].

Several reports indicate that HLB-infected trees require higher nutrient levels compared to those recommended for healthy plants [26,28,29]. However, other studies suggest a greater nutrient uptake efficiency in HLB-infected plants than in healthy ones [30]. Notably, not many comparisons have been made considering citrus plants with different levels of HLB tolerance. Ghimire et al. [29] assessed the nutrient uptake potential in HLB-tolerant and -susceptible rootstocks. Tolerant rootstock A + Volk × O-19 had the highest nutrient uptake efficiency, while susceptible US-896 exhibited lower nutrient uptake efficiency and a reduced expression of nutrient transporter genes.

Additional studies highlighted differences in the effectiveness of micronutrient application methods, such as granular versus liquid forms, where micronutrient application rates did not correlate with leaf micronutrient concentrations [30]. Additional research is necessary to better understand the nutrient uptake capacity of Persian lime across different rootstocks under *C*Las infection. This information will assist in designing improved fertilization programs [29].

Applying the correct fertilizer at the right time, in the proper amount and location, and from a suitable source will enhance the effectiveness of fertilization programs [31,32]. Recently, a consistent and gradual application of fertilizers has been adopted for managing HLB in Florida, USA [33,34]. This approach reaches the crop’s annual fertilizer requirement through smaller doses administered more frequently rather than all the fertilizer at once. The plant obtains a continuous supply of nutrients, avoiding leaching losses and preventing nutrient deficiencies associated with HLB [32]. Fertigation allows for up to thirty applications each year, whereas growers who depend on rainfed conditions typically have four to six fertilizations each year [33,34].

Identifying the tolerance characteristics of Persian lime is crucial for determining molecular targets that will help develop effective disease management strategies. This information will assist in implementing a balanced fertilization program specifically designed for Persian lime affected by HLB. Balanced fertilization strategies are necessary for practical field applications under irrigation or rainfed conditions. Recent progress has been made in understanding the role of macro- and micronutrients, as well as their interactions and effects on citrus trees affected by HLB, particularly in *C. sinensis* [26,33,35,36,37]. However, in Mexico, the impact of balanced fertilization has not been thoroughly studied in either tolerant or susceptible citrus species. This highlights the need for developing an HLB management strategy for Persian lime based on local research, to design short-term, applicable management strategies. This study focuses on nitrogen (N), phosphorous (P), and potassium (K) as key macronutrients, complemented by calcium (Ca), magnesium (Mg), and sulfur (S) as secondary macronutrients, as well as zinc (Zn), boron (B), manganese (Mn), and iron (Fe) as micronutrients. Strategies with different levels of macro- and micronutrients were designed based on recommended regional doses and commercial fertilizers used locally. We hypothesize that applying macronutrients and micronutrients in split doses through soil and/or foliar methods can enhance nutrient efficiency. This approach may reduce the amounts needed while improving the physiological and hormonal responses of *C. latifolia* plants infected with *C*Las. To test this hypothesis, we compared the effects of low, medium, and high doses of individual macronutrients, as well as their combination with micronutrients, using soil and/or foliar application methods. We assessed their impact on foliar symptoms, *C*Las populations in midribs, and various physiological parameters, including growth and development, photosynthetic rate, leaf spectral reflectance, vascular bundle damage, carbohydrate metabolism, and the accumulation of salicylic acid (SA) and its biosynthetic precursors. This approach will help to develop new tools for Huanglongbing disease management in Persian lime.

## 2. Results

### 2.1. Relative Quantification of CLas 16S rDNA Gene After Fertilization Treatments

**Figure 1:**
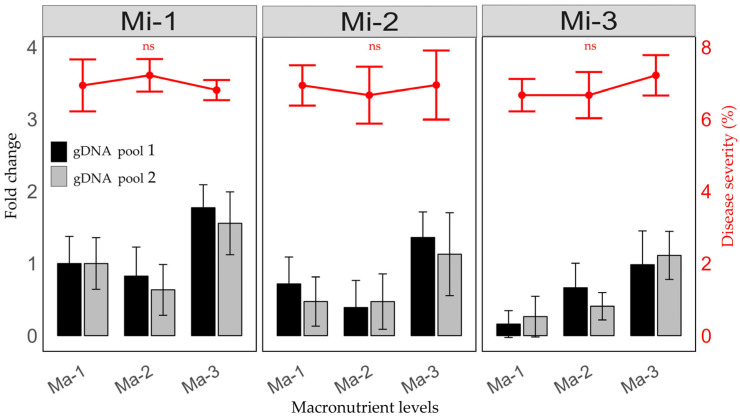
Change in the number of bacteria for high and medium fertilization treatments compared to the Ma-1 + Mi-1 treatment and its relationship with the severity of symptoms on leaves. Ma = macronutrients, Mi = micronutrients. Mean ± propagated error, ns = not significant.

The amplification of the 16S rDNA gene at the end of summer assessed the change in the relative accumulation of *C*Las. This variable evaluated the effect of medium and high macro- and micronutrient treatments compared to the Ma-1 + Mi-1 treatment. No increase in the number of bacteria (with a value equal to or less than 1) was observed for the treatments with low and medium doses of macronutrients (Ma-1 and Ma-2) across all micronutrient levels (Mi-1, Mi-2, and Mi-3). In contrast, an increasing trend was observed with high doses of macronutrients at all three micronutrient levels (Figure 1), especially with the absence of micronutrients (Ma-3 + Mi-1).

### 2.2. Fertilization and the Percentage of Severity of HLB Symptoms in Leaves

At the end of the experiment, no significant differences were observed in the percentage of severity among all fertilization treatments (*p* = 0.4728) (Figure 1). The percentage of severity did not exceed 8.0% for any treatment; this rate was lower than results reported for susceptible varieties and those observed in field conditions for Persian lime. Thus, balanced fertilization was effective in delaying the progression of symptoms.

### 2.3. Fertilization on the Efficiency of Photosystem II (Fv/Fm) in Healthy and Diseased Plants

**Figure 2:**
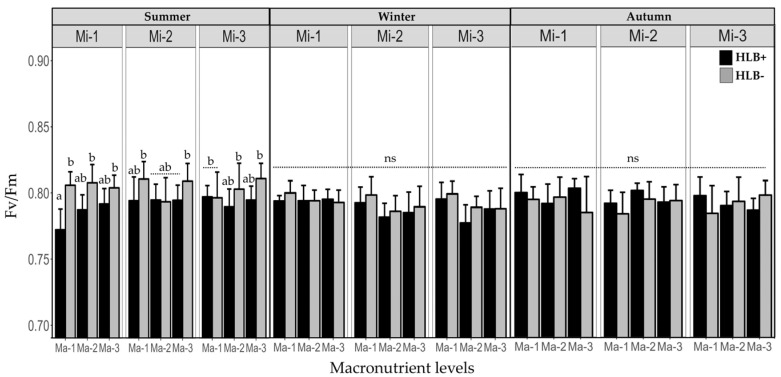
Effect of *C*Las infection and macronutrient and micronutrient levels on the efficiency of photosystem II. ns = not significant. Different letters indicate significant differences (Tukey, *p* < 0.05).

Before treatment applications, variation in Fv/Fm values was noted among the plant groups. During the summer, means of Fv/Fm were lower in diseased plants (0.791 ± 0.008) than in healthy plants (0.80 ± 0.006) (*p_s_* ≤ 0.0001). However, after fertilization treatments were applied, Fv/Fm values did not differ significantly between healthy and HLB-infected plants in the winter and autumn (*p_s_* = 0.1260 and 0.3689, respectively) (Figure 2).

### 2.4. Reflectance Spectrum on Leaves of Healthy and HLB-Diseased Plants After Fertilization Treatments

**Figure 3:**
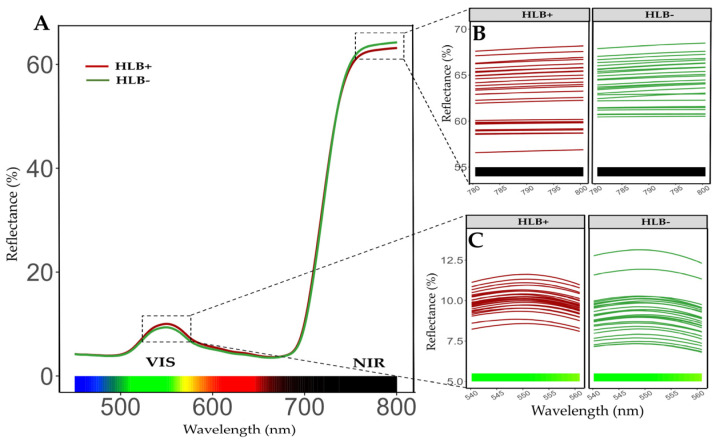
(**A**) Spectrograms with means of reflectance of leaves from healthy (green line) and diseased (red line) plants. (**B**,**C**) Wavelengths of 550 nm in the visible region (VIS) and 790 nm in the near-infrared (NIR) regions were chosen to analyze reflectance on plants treated with different fertilization strategies.

Mean reflectance data collected during the experimental period revealed notable differences between healthy and diseased plant groups in both the visible light spectrum (VIS) and near-infrared (NIR) regions (Figure 3A). Wavelengths of 550 nm (visible light) and 790 nm (near-infrared) showed the most significant variability between groups. The first one may have been related to the loss of photosynthetic pigments and the last one to the accumulation of carbohydrates, due to *C*Las infection (Figure 3B,C). Diseased plants had a higher percentage of reflectance at 550 nm compared to healthy plants. Conversely, at 790 nm, the response was the opposite. Unlike the trend in photosynthetic efficiency, at the beginning of the experiment (summer), no differences were observed between treatments in the VIS range (*p_Ma×Mi_* = 0.7194). These results contrast with those obtained during the winter (*p_Ma×Mi_* = 0.0021) and autumn, where only macronutrient levels had a marginally significant effect (*p_Ma_* = 0.0589, *p_Ma×Mi_* = 0.5316) (Figure S1A). In the NIR range, no significant differences were found in the summer, winter, or autumn (*p_Ma×Mi_* = 0.8634, 0.3083, and 0.2585, respectively) (Figure S1B). A significant variation in the percentage of reflectance was observed between healthy and diseased leaves. Additionally, no clear correlation was found between the percentage of reflectance and fertilization treatments.

### 2.5. Fertilization Impact on the Content of Chlorophyll, Sucrose, and Starch in Healthy and HLB-Diseased Leaves

**Figure 4:**
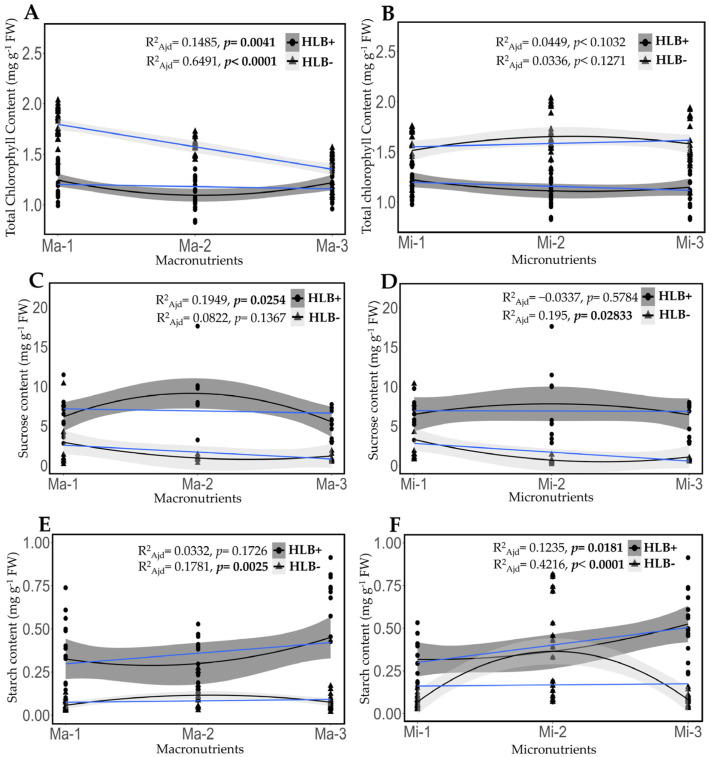
Polynomial regression analysis for macronutrient (**A**,**C**,**E**) and micronutrient (**B**,**D**,**F**) treatment levels and their effect on total chlorophyll, sucrose, and starch content in healthy and HLB-diseased plants. The blue line represents the data linear fit. Values represent four replicates, mean ± standard deviation. Ma = macronutrients, Mi = micronutrients. Significant *p*-values are shown in bold.

At the end of the experiment, diseased plants were affected in all variables of carbohydrate and chlorophyll metabolism. Healthy plants showed 24.2% more chlorophyll (1.57 mg g^−1^ FW ± 0.20 SD) compared to diseased plants (1.19 mg g^−1^ FW± 0.11 SD). Also, healthy plants presented 75.4% less sucrose concentration (1.68 mg g^−1^ FW ± 2.18 SD) and 80.0% less starch (0.08 mg g^−1^ FW ± 0.03 SD) compared to diseased plants (sucrose: 6.87 mg g^−1^ FW ± 2.39 SD; starch: 0.41 mg g^−1^ FW ± 0.20 SD) (Figure S2A–C). Also, starch accumulation and several anatomical changes in the central vein of the leaves were observed in diseased plants compared to healthy ones (Figure S3). Results suggest that HLB causes damage to the phloem and alters carbohydrate metabolism. Low doses of macronutrients (Ma-1) were associated with the highest chlorophyll content and the lowest sucrose and starch contents in leaves of HLB-diseased Persian lime plants. In contrast, higher doses of macronutrients showed reduced chlorophyll content in healthy plants and a more significant starch accumulation in diseased plants (Figure 4A,C,E). The micronutrient levels applied did not have a significant effect on chlorophyll content in healthy and HLB-infected plants (*p* = 0.1271 and 0.1032, respectively). Micronutrients influenced sucrose content in healthy plants (*p* = 0.0283) but not in infected plants (*p* = 0.5784). The highest starch accumulation in diseased plants occurred with soil and foliar application (Mi-3) (*p* = 0.0181) and with soil applications in healthy plants (Mi-2) (*p* = 0.0001) (Figure 4B,D,F).

### 2.6. Fertilization Impact on Cumulative Growth in Height, Canopy Volume, and Trunk Diameter in Healthy and HLB-Diseased Plants

Infection with *C*Las significantly affected cumulative plant growth and canopy volume and fostered a larger trunk diameter in healthy plants (*p_s_* ≤ 0.0001 for all three variables) (Figure S4A–C).

On average, healthy plants showed a 17.0% additional cumulative height (132.58 cm ± 14.86 SD) compared to that in diseased plants (109.03 cm ± 12.04 SD). They also showed 33.7% more canopy volume (2.40 m^3^ ± 0.33 SD) than diseased plants (1.59 m^3^ ± 0.34 SD), while having a 19.5% smaller trunk diameter (9.44 mm ± 2.11 SD vs. 11.72 mm ± 2 SD in diseased plants). This difference might be attributed to an increase in the phloem area of the stem, similar to that observed in the midrib of leaves (Figure S4A–C).

**Figure 5:**
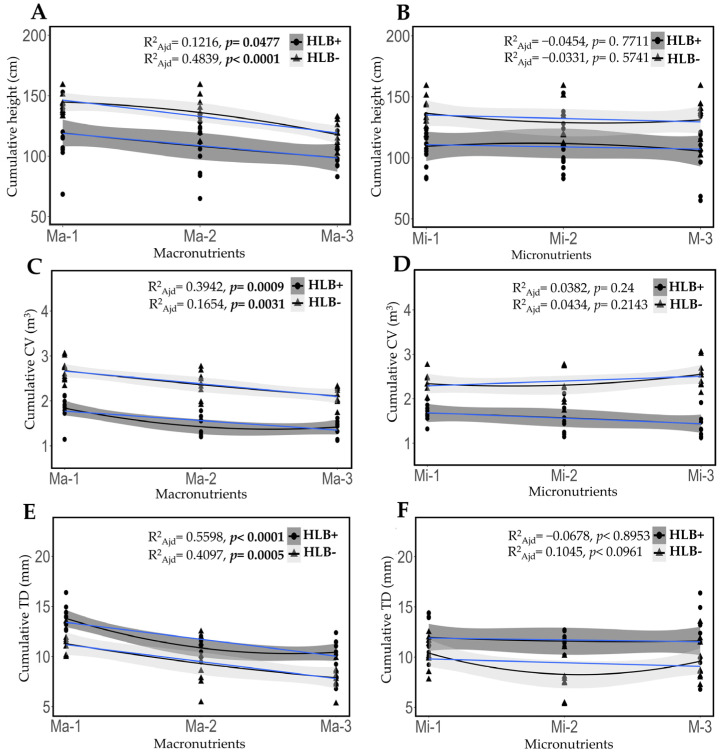
*C*Las infection and fertilization treatments had an impact on the growth and development of Persian lime plants. Polynomial regression analysis was conducted to evaluate the effects of macronutrient (**A**,**C**,**E**) and micronutrient treatment levels (**B**,**D**,**F**) on cumulative height, canopy volume, and trunk diameter in both healthy and HLB-diseased plants. The blue line indicates the linear fit of the data. Values are expressed as mean ± standard deviation. Ma = macronutrients, Mi = micronutrients, CV = canopy volume, TD = trunk diameter. Significant *p*-values are shown in bold.

After fertilizing every 18 days over the span of one year, the lowest doses of macronutrients were associated with the highest cumulative growth, greater canopy volume, and trunk diameter in healthy and diseased plants (Figure 5A,C,E). In contrast, micronutrient levels showed no significant effect in healthy or diseased plants (Figure 5B,D,F). Increased doses of macronutrients plus the application of micronutrients, both to the soil and foliar, do not seem to be favorable for promoting the growth and development of HLB-infected Persian lime plants.

### 2.7. Fertilization Impact on Endogenous Content of Salicylic Acid and Its Biosynthetic Precursors in Healthy and HLB-Diseased Plants

**Figure 6:**
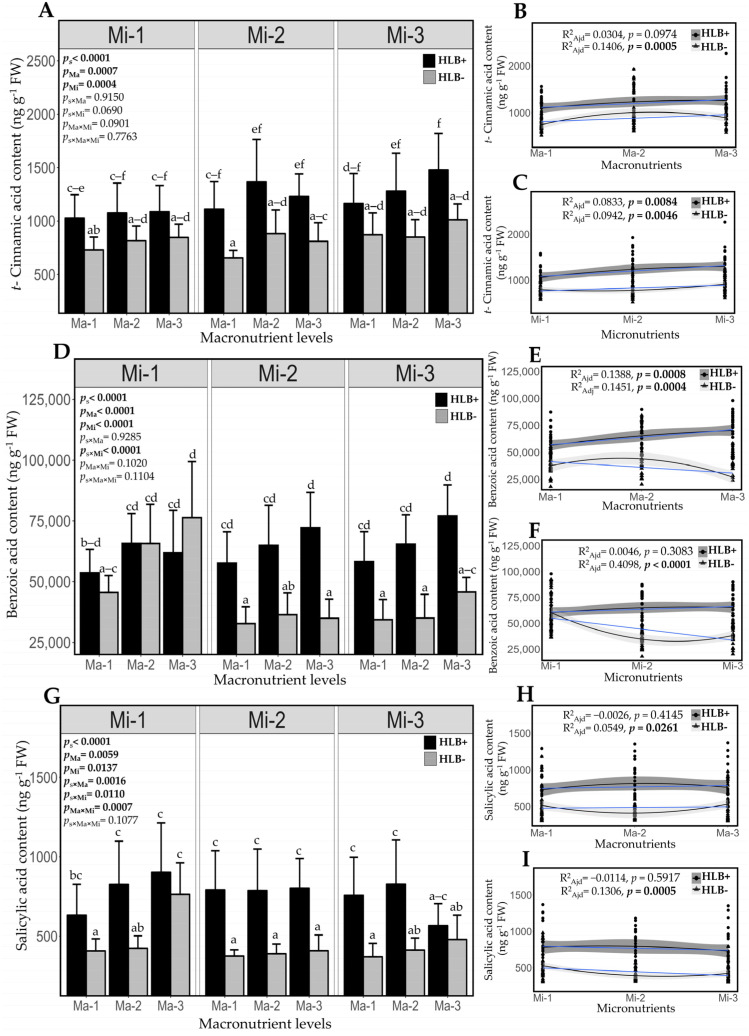
Impact of *C*Las infection and fertilization treatment on the content of endogenous salicylic acid and its biosynthetic precursor content. (**A**,**D**,**G**) Concentration of *trans*-cinnamic acid, benzoic acid, and salicylic acid in leaves (**B**,**C**,**E**,**F**,**H**,**I**). Polynomial regression analysis for macro- and micronutrient levels in healthy and diseased plants. The blue line represents the linear fit of data. According to the general linear model (ANOVA), the main effects are shown within graphs. Different letters indicate significant differences (Tukey, *p* < 0.05). Values represent mean ± standard deviation. Ma = macronutrients, Mi = micronutrients. Significant *p*-values are shown in bold.

*C*Las infection increased the endogenous content of *t*-CA, BA, and SA in diseased plants compared to healthy plants. Specifically, healthy plants showed 30.9% lower *t*-CA levels (830.55 ng g^−1^ FW ± 99.84 SD) than diseased plants (1202.15 ng g^−1^ FW ± 149.80 SD). In addition, healthy plants had 29.5% lower BA levels (45,218.49 ng g^−1^ FW ± 15,608.23 SD) than diseased plants (64,104.37 ng g^−1^ FW ± 7315.19 SD). Furthermore, salicylic acid (SA) levels were 41.5% lower in healthy plants (447.64 ng g^−1^ FW ± 122.69 SD) compared to diseased plants (765.72 ng g^−1^ FW ± 103.52 SD) (Figure 6A,D,G).

Consistent with the results above, the lowest doses of macronutrients were associated with the lowest concentrations of *t*-CA and BA in diseased plants, with a trend to increase at higher doses. A similar trend regarding *t*-CA was observed in healthy plants; however, higher doses were associated with the lowest concentration of BA. SA concentration was similar at all macronutrient levels in diseased plants, while in healthy plants, it was lower at the medium macronutrient dose (Figure 6B,E,H). Micronutrient deficiency was associated with lower *t*-CA concentrations in healthy and diseased plants. Furthermore, micronutrient application to the soil resulted in the lowest concentration of BA and SA in healthy plants. On the other hand, the concentration of these analytes was similar in diseased plants, regardless of micronutrient levels (Figure 6C,F,I). Healthy and HLB-infected plants exhibit different hormonal responses, indicating that nutrition may affect hormone regulation and the Persian lime’s ability to respond to HLB disease progression.

### 2.8. Variables Associated with Healthy and HLB-Diseased Plant Groups Obtained by Principal Component Analysis (PCA)

The contributions of PC1, PC2, and PC3 were 39.73%, 12.26%, and 9.90%, respectively, accounting for 61.89% of the total variance in the data. The principal components plot showed a clear separation between healthy and diseased plants, indicating differences in the linear properties between the study variables. Healthy plants were associated with higher values for height, canopy volume, photosynthetic efficiency, chlorophyll, and reflectance at 790 nm. In contrast, diseased plants showed elevated values for trunk diameter, glucose, sucrose, starch, *trans*-cinnamic acid, benzoic acid, salicylic acid, and reflectance at 550 nm (Figure S5). These findings suggest that HLB significantly affected most of the variables studied, particularly those linked to growth and development, carbohydrate metabolism, reflectance, and hormone levels, despite the fact that Persian lime is generally considered tolerant to this disease.

## 3. Discussion

As a species tolerant to HLB, Persian lime shows symptoms more gradually, which enables it to sustain development, cellular homeostasis, and productivity even when infected [13,38]. The tolerance observed in specific citrus cultivars is associated with polyploidy in both rootstocks and commercial varieties, contributing to more efficient detoxification and enhanced phloem regeneration upon *C*Las infection [14,39]. In this case, the severity of HLB symptoms in leaves remained below 8.0% in all treatments almost two years after the initial inoculation. However, a severity percentage greater than 40.0% has been reported in Persian lime trees in the field [40], suggesting that fertilization may have contributed to the slow development of leaf symptoms.

*C*Las infection affects chlorophyll fluorescence parameters, including photosystem II efficiency (Fv/Fm) [41]. While diseased plants exhibited a lower total chlorophyll content than healthy plants, their photosynthetic efficiency remained similar across both groups. This finding aligns with the low severity percentage observed in the leaves. Reduced photosynthetic efficiency in diseased plants is linked to increased damage to the photosynthetic apparatus caused by HLB infection [41]. However, fertilization can enhance photosynthetic efficiency by reducing photoinhibition caused by stress [42]. Low doses of macronutrients seem to support stability in chlorophyll content and photosynthetic efficiency. This aligns with previous studies demonstrating how proper fertilization can enhance the chlorophyll index in leaves and promote greater photosynthetic capacity [43]. The decline in chlorophyll content of the leaves in diseased plants was less pronounced than what was reported in symptomatic trees in the field [44]. The loss of chlorophyll may occur due to the buildup of carbohydrates, such as sucrose and glucose, which can suppress certain genes involved in photosynthesis [45]. Furthermore, the buildup of starch in mesophyll chloroplasts can lead the rupture of thylakoids and the degradation of chlorophyll [46].

The blockage of photoassimilate flow in the phloem due to HLB results in an accumulation of carbohydrates in the leaves, including glucose, fructose, sucrose, and starch [47]. This accumulation can affect photosynthesis and reflectance parameters by altering leaf pigments, their biochemical composition, and cellular structure [48]. Although Persian lime shows some tolerance to HLB, research indicates that diseased plants accumulate more glucose, sucrose, and starch in their leaves than healthy plants. This finding confirms that the vascular system of Persian lime is affected by HLB, but to a lesser extent than in more susceptible citrus varieties and species. This buildup of carbohydrates occurs due to the reduced flow of photoassimilates caused by the obstruction of phloem sieve plate pores. This phenomenon was previously observed in diploid varieties, such as the Mexican lime, and triploid varieties, such as the Persian lime. However, reports indicate that Persian lime presents less collapse of phloem cells, as well as an increase in the phloem area of the petioles [14]. This observation aligns with our findings in the leaf veins (Figure S3).

Leaf reflectance is affected by several characteristics, such as the surface, internal structure, and biochemical composition of the leaf [49,50]. Healthy and HLB-diseased Persian lime plants showed distinctive reflectance patterns in the VIS and NIR, consistent with previous reports on reflectance assessments in citrus [48,51]. However, the mild severity of symptoms caused significant variation among plants and sampling times. Still, PCA showed that reflectance at 550 nm is a variable associated with diseased plants, even with disease management actions, which could further mask symptom expression in Persian lime. Although the equipment is capable of distinguishing between healthy and diseased plants, the variation between asymptomatic leaves and those with mild HLB symptoms poses a challenge for early detection. This issue is particularly important for devices that operate solely within the visible light spectrum (400–800 nm) and lack trained software to improve detection accuracy. More advanced reflectance methods could improve efficiency in detecting HLB. Hu et al. [52] combined multicolor fluorescence images with multispectral reflectance and a pre-trained convolutional neural network model, which allowed them to detect HLB in the field with an efficiency greater than 96.0%.

Diseased Persian lime plants showed lower cumulative growth in height and canopy volume, as well as a greater trunk diameter compared to healthy plants. The adverse impact of the disease on the height and canopy volume of affected plants has been well documented, alongside the beneficial effects of foliar micronutrients [35,53]. Our results are aligned with these findings, where the mildest impact on growth variables in diseased plants occurred when low doses of macronutrients were combined with micronutrients in the soil, in contrast to higher doses. Although the increase in trunk diameter in plants infected with HLB has not been previously reported, some studies indicate anatomical damage to the phloem due to the effect of HLB [54,55]. In addition, an increase in phloem thickness has been reported in Persian lime, attributable to more significant regeneration in response to HLB damage, which appears to be one of the tolerance characteristics provided by polyploidy [14].

Phytohormones are crucial in regulating plant growth and development as well as in the signaling networks that coordinate most physiological functions in plants, including the response to pathogens [56]. This work evaluated the endogenous expression of salicylic acid and its biosynthetic precursors in the phenylalanine ammonia-lyase (PAL) pathway, *trans*-cinnamic acid, and benzoic acid. The results showed a higher concentration of *t*-CA, BA, and SA in diseased plants compared to healthy ones, consistent with previous reports in healthy [57] and diseased [58] orange plants. However, there are no reports of the hormonal response of Persian lime to *C*Las infection. Additionally, the quantification of these analytes in Persian lime leaves revealed lower concentrations of *t*-CA and BA at the lowest doses of macronutrients, and these levels have an upward trend at higher doses. This finding suggests a more significant stress condition at the highest doses, which aligns with alterations in other growth variables, the carbohydrate metabolism, chlorophyll content, and bacterial presence observed by the end of the experiment.

Previous research has shown differences in the phytohormones pathways, such as SA, between tolerant and susceptible citrus varieties [11,46,59,60]. Higher concentrations of SA and *Me*-SA were reported in tolerant grapefruit (*C. paradisi* Macf.) compared to susceptible *C. sinensis*. However, after inoculation with *C*Las, SA levels were reduced to less than half in grapefruit, while SA concentration in susceptible oranges increased [59]. Similarly, Suh et al. [11] found that metabolites related to the SA signaling pathway decreased in tolerant Sugar Belle^®^ mandarin compared to susceptible Murcott mandarin. In *Citrus reticulata* Blanco, a moderately tolerant species, *C*Las infection repressed the SA and ethylene signaling pathway [60]. On the other hand, Curtolo et al. [46] did not report changes in the transcription of genes related to SA biosynthesis in *P. trifoliata*. The results suggest that the SA-mediated defense response could be suppressed in tolerant varieties upon *C*Las infection.

Although in this work, SA levels increased upon *C*Las infection, the concentration found in HLB-tolerant Persian lime leaves was lower than that reported in susceptible varieties [13]. The increase in BA levels could be associated with the increase in SA levels [61], which differs from our results and leads us to hypothesize a lower conversion of BA to SA. SA synthesis occurs via two chorismate-dependent pathways in the shikimic acid pathway. The first converts chorismate to isochorismate via isochorismate synthase (ICS) and then to SA via isochorismate pyruvate lyase (IPL) [62]. The second pathway is through PAL, which converts L-phenylalanine to cinnamic acid, which is converted into benzoic acid by aldehyde oxidase (AO) and finally into SA via benzoic acid-2-hydroxylase (BA2H) [61,62]. No homologous genes for IPL or BA2H have been found in citrus, suggesting that, in this case, the PAL-dependent pathway could be responsible for SA biosynthesis [58,63]. This statement accounts for the increased concentration of BA observed in Persian lime; however, it does not explain the reduced concentration of SA. All these ideas suggest a lower conversion rate of BA to SA, both in healthy and diseased plants, which may indicate that Persian lime exhibits some tolerance to HLB. Furthermore, we found a significant positive correlation between BA and SA content in healthy plants (R^2^ = 0.54) but not in diseased plants (R^2^ = 0.07). A possible characteristic of tolerant varieties is the coexistence with the disease, either restricting or delaying the defense response, thereby reducing damages caused by the disease and supporting the hypothesis that HLB is an autoimmune disease [12]. The hypothesis of SA biosynthesis via the PAL pathway has been raised based on the results in varieties susceptible to HLB; however, citrus species such as *C. aurantifolia* and *C. latifolia* have shown lower PAL activity than *C. sinensis* [64], which may indicate a reduced PAL-dependent pathway activity in HLB-tolerant varieties.

Overall, the analysis of the combination of macronutrient and micronutrient levels suggests that the low doses of macronutrients used in the present study, combined with soil application of micronutrients (Ma-1 + Mi-2), allow diseased plants to show greater height, canopy volume, and chlorophyll concentration, as well as less starch accumulation in leaves. The results with lower-than-recommended doses agree with previous reports, where a potential reduction in N dose of up to 25.0% does not affect vegetative growth, juice yield, and juice quality in HLB-diseased citrus [65]. They also support the relieving effect of micronutrient application to HLB-diseased plants [24,35].

*C*Las infection induces phosphorus deficiencies in *C. sinensis* due to the overexpression of microRNAs, such as miR399, which encodes a ubiquitin-conjugating enzyme (PHO2) related to the degradation of phosphorus-transporting proteins [66]. A subsequent study confirmed that infection with *C*Las has a significant effect on the efficiency of phosphorus resorption; however, in tolerant varieties such as *C. limon* and *C. maxima*, infection with *C*Las did not affect phosphorus levels in leaves, unlike what was observed in HLB-susceptible *C. reticulata* [67]. On the other hand, reports indicate that diseased plants are more efficient in nutrient uptake, despite the reduction in root volume due to the effect of HLB. This is attributed to the overexpression of genes related to the transport of cations such as NH_4_^+^, K^+^, Zn^2+^, Fe^2+^, and Cu^2+^ [43].

The results indicate that the increase in macronutrient doses, along with the simultaneous soil and foliar application of micronutrients in short periods, reduces the efficiency of the applications, possibly due to the excessive presence of these micronutrients. This effect was reflected in growth, carbohydrate metabolism, and an increase in the number of bacteria. In addition, greater fertilizer efficiency was observed by applying lower doses, split into more applications per year, compared to the higher doses evaluated, also with a split application. Constant fertilization improves the availability and uptake of nutrients by citrus plants that have lost secondary roots due to the disease; it also reduces leaching losses [23]. Split fertilizer application was evaluated in citrus trees with HLB, and the results have shown an improvement in canopy volume and a reduction in nitrogen requirement with 20 applications per year, in addition to improving the nutritional content at the foliar level and the nitrogen content in the soil in the form of nitrates and ammonium [25]. These results require field validation, particularly with bearing trees, to determine the appropriate dose and number of annual applications in Mexico’s citrus-growing areas, complemented with cost–benefit analysis. Some studies have evaluated the economic feasibility of enhanced nutritional programs, with contradictory results. While some authors report benefits in yield and profitability, others have found no significant advantages [68,69]. Ozores-Hampton et al. [70] documented that an enhanced foliar nutrition program applied over three years increased yield by 7.0 to 9.0% in Valencia oranges affected by HLB. In contrast, Bassanezi et al. [71] evaluated various fertilization programs in Valencia oranges with HLB. Surprisingly, the standard nutritional program used by growers provided the best cost–benefit ratio, requiring fewer foliar applications than other treatments. This result aligns with our findings regarding the continuous application of both soil and foliar fertilizers, which does not appear to be beneficial for both healthy and HLB-affected Persian lime trees. Additionally, the economic feasibility of large-scale split applications in tolerant varieties such as Persian lime are needed. Implementing this strategy in rainfed orchards might increase labor costs. However, reducing the total amount of fertilizers could help offset the increased costs.

## 4. Materials and Methods

### 4.1. Study Site Description and Plant Material Used

The study was conducted in a greenhouse (290 m^2^) in Central Veracruz, Mexico (Lat. 19.194167, Long −96.343611). Persian lime (*Citrus latifolia* Tan.) plants, grafted onto 2-year-old macrophylla (*Citrus macrophylla* Macf.) rootstocks, were purchased from a certified nursery. Plants were transferred to a 40 × 40 cm container with a 2:1 mixture ratio of soil (pH: 5.75 ± 0.07, texture: sandy loam, organic matter: 16.7%, electrical conductivity: 1.9025 dS/m, phosphorus content: 475 mg L^−1^, potassium content: 700 mg L^−1^, nitrite content: 2 mg L^−1^, nitrates content: 500 mg L^−1^) and Peat moss^®^. A group of plants were grafted at the end of summer with buds from a tree confirmed positive for *C*las by PCR, originating from plots of the National Institute of Forestry, Agricultural, and Livestock Research (INIFAP), Ixtacuaco Experimental Station. Both healthy and infected groups were grafted in November 2020. A healthy group of plants was grafted with buds produced in the certified nursery. The seasonal variability in macro- and micronutrient demand was associated with Persian lime growth cycles. Therefore, the experiment was conducted over a full year, starting in the summer of 2021 and ending in autumn in 2022.

### 4.2. Fertilization Treatments

This study evaluated three levels of macronutrients, low (Ma-1), medium (Ma-2), and high (Ma-3), and three conditions for micronutrient application: no additional application (Mi-1), soil-applied (Mi-2), and soil- and foliar-applied (Mi-3) (Table S1). Macronutrient treatments included N, P, and K as essential elements, with Ca and Mg added in medium (Ma-2) and high (Ma-3) doses. Soil-applied micronutrients (S, Zn, B, Mn, and Fe) were selected for their essential roles and potential benefits in enhancing the health of HLB-affected citrus trees [23,37,72]. The medium macronutrient dose was formulated based on regional recommendations designed by INIFAP to meet the nutritional requirements of two-year-old plants [73]. The low macronutrient dose (Ma-1) was defined as half of the medium dose (Ma-2), while the high dose (Ma-3) was determined based on the nutrient requirements of three-year-old plants. The amount of soil-applied micronutrients at medium and high doses of macronutrients was calculated based on the recommendations of Morgan and Kadyampakeni [34] for non-bearing trees, taking into account the amount of nitrogen needed. The treatment with foliar-applied micronutrients associated with low doses of NPK (Ma-1 + Mi-3) consisted of 1.1% B, 1.3% Zn, 6% Fe, 2.4% Mn, 0.25% Cu, and 0.25% Mo. Foliar-applied micronutrient treatments at medium and high macronutrient doses (specifically Ma-2 + Mi-3 and Ma-3 + Mi-3) contained 4.8% N, 4.9% Mg, 4.9% B, and 9.9% Zn. Micronutrient concentrations used in foliar treatments followed recommendations of commercially available products. Commercial granulated fertilizers were used for all soil treatments, while both soil and foliar applications were carried out every 18 days, according to the recommendations of Atta et al. [74].

The experiment used a 2 × 3 × 3 factorial design with two health levels (healthy and diseased plants), three macronutrient levels, and three micronutrient levels. A randomized block design was used for analysis, with nine fertilization treatments applied to both infected and non-infected plants, with four replicates per treatment. Plants were placed in the greenhouse in late July 2021, with an initial spacing of approximately 1 m. Distance increased as required by canopy volume.

### 4.3. Severity of Foliar Symptoms

The damage scale proposed by Gottwald et al. [75] was used to evaluate foliar symptoms. Each tree was divided into eight sections, individually assessed for severity on a scale of 0 to 5, based on the proportion of leaves showing HLB symptoms. The sum of the results of each section resulted in a range of severity per tree with values from 0 to 40. To standardize assessment criteria, photographs displaying the characteristic symptoms of HLB were printed. The percentage of severity for each tree was calculated using the Shokrollah et al. [76] formula:

(1) $$\mathrm{Disease}\;\mathrm{severity}=\frac{\sum \left(a\times b\right)}{N\times Z}\times 100$$

where $\sum \left(a\times b\right)$ = sum of the symptomatic plant and their corresponding rating, *N* = total number of sampled plants, and *Z* = highest rating.

### 4.4. DNA Extraction and CLas Detection

DNA extraction was performed using the CTAB protocol in March–April 2021 [77]. Midribs of four leaves per plant were collected from the middle section of the canopy. Confirmation of healthy and diseased plants at the start of the experiment was determined by PCR, amplifying the 16S gene (rDNA) of *C*Las with the enzyme MyTaqTM DNA Polymerase (Bioline^®^, London, UK) and oligos OI/OI2c. PCR protocol started at 95 °C for 5 min, followed by 35 cycles of 30 s at 94 °C, 30 s at 62 °C and 1 min at 72 °C, with a final extension of 10 min at 72 °C according to Jagoueix et al. [78].

At the end of the experiment, the gDNA from the four plants in each treatment was equimolarly mixed, and qPCR amplifications were conducted according to the recommendations of Li et al. [79], with some modifications. Specific primers, *C*Las-4G [80], HLBr, and the HLBp probe were used to amplify the 16S gene of *C*Las. Additionally, primers COXF, COXR, and the COXp probe were used to quantify the endogenous COX gene [79]. Two replicates were run for each gDNA pool, and every reaction was performed in triplicate using the Taq PCR Master Mix Kit (Qiagen^®^, Hilden, Germany). The Ct obtained in qPCR reactions was used for the relative quantification of the *C*Las genes, following the 2^−∆∆Ct^ method [81]. The relative quantification of the 16S rDNA gene of *C*Las was assessed at the end of the experiment (autumn 2022). Changes in the amount of *C*Las under the fertilization treatments were compared to those of the Ma-1 + Mi-1 treatment. The propagated error of the ∆CT values for the 16S and COX genes was calculated [81].

### 4.5. Growth Variables

The trunk diameter was measured using a digital vernier caliper at a height of 15 cm from the base of the stem. Canopy volume (CV) was determined by assessing the canopy diameter in both the east–west and north–south directions, considering plant height. Then, the prolate spheroid formula VD (m^3^) = (4/3) × (π) × (height/2) × (average canopy radius)^2^ was used [82]. Measurements were taken monthly over the course of one year. Net growth (in cm) per month was calculated by subtracting the previous month’s value from the value obtained in the current month for the three parameters evaluated. Monthly values were added, with the final month’s value considered as the accumulated growth in height, canopy volume, and trunk diameter.

### 4.6. Photosystem II (Fv/Fm) Efficiency

Maximum photosystem II efficiency (Fv/Fm) was measured in healthy and diseased Persian lime leaves using a portable OS30p fluorometer (Opti-Sciences) during summer 2021, winter 2022, and autumn 2022. The leaves from healthy and diseased plants were adapted to dark conditions before measurements. Data collection occurred under daylight conditions in the greenhouse at a consistent morning time.

### 4.7. Chlorophyll Quantification in Leaves

The methodology outlined by Das et al. [83] was applied for chlorophyll extraction, incorporating modifications from Flores-de la Rosa et al. [44]. The absorbance of the solution was measured at wavelengths of 645 and 633 nm using a spectrophotometer, with acetone serving as a comparative blank. Total chlorophyll content was calculated using the following formula [84]:

mg of total chlorophyll/g of tissue = [20.2 (A645) + 8.02 (A633)] × V/1000 × FW(2)

where A = absorbance at a specific wavelength (633 and 645 nm), V = final volume of chlorophyll extracted in 80% acetone, and FW = sample fresh weight.

### 4.8. Quantification of Sucrose and Starch in Leaves

Sucrose extraction was carried out at the end of the experiment (autumn 2022) using the methodology described by Geigenberger and Stitt [85]. For quantification, commercial kit SCA20 (Sigma-Aldrich^®^) was used. A calibration curve was constructed with pure rice starch to evaluate starch (y = 0.0011x + 0.0029, R^2^ = 0.9997). The extraction was conducted following the methodology outlined by Zheng et al. [86], with modifications made by Flores-de la Rosa et al. [44]. Sucrose was quantified by measuring the absorbance at 594 nm.

### 4.9. Reflectance Measurement Using Spectrometry

A spectrometer with a spectral range of 339 to 822 nm and a resolution of 0.45 nm was used to assess changes in leaf reflectance, resulting from the loss of photosynthetic pigments. Based on preliminary results, analyses were focused on reflectance at 550 nm (VIS) and 790 nm (NIR) (Figure 3). The QP600-025-VIS optical fiber is connected to a 74-UV series collimating lens, positioned 5 cm from the module’s base. Oceanview^®^ software (version 2.0.8) helped to capture data. Spectrograms were generated using the ggplot2 package in RStudio (v4.3.1, R Project). The reflectance module was built under dark conditions, with two 90 w halogen lamps serving as the primary light source. A white polytetrafluoroethylene panel was used to calibrate the equipment. Results were analyzed by building spectrograms illustrating the percentage of reflectance of healthy and diseased plants. Reflectance data were collected in summer 2021, winter 2022, and autumn 2022.

### 4.10. Quantification of Benzoic Acid, Trans-Cinnamic Acid, and Salicylic Acid in Leaves Using GC-MS

To evaluate the hormonal response of healthy and HLB-diseased plants, the accumulation of salicylic acid (SA) and two precursors, benzoic acid (BA) and *trans*-cinnamic acid (*t*-CA), in leaves was quantified. Gas chromatography/mass spectrometry (GC-MS) was used at the end of the experiment (autumn 2022), following the methodology reported by Hijaz et al. [87] and modified by Nehela et al. [57]. Derivatization of the standards was performed using the same method. Calibration curves were generated by injecting 1 µL of pure standards at concentrations of 100, 50, 20, 10, 5, 2, and 1 ng µL^−1^ for BA, and 10, 5, 2, 1, 0.5, and 0.2 ng µL^−1^ for both SA and *t*-CA. The GC-MS was operated in SIM and Scan modes. Quantitative and qualitative ions were monitored for each standard. Results were compared with the NIST (National Institute of Standards and Technology) library using the MassHunter^®^ program. Validation was conducted by assessing specificity, linearity, precision, accuracy, detection, and quantification limits, following Rawlinson et al.’s [88] criteria (Table S2).

### 4.11. Statistical Analysis

The analysis was conducted using RStudio version 4.3.1 [89]. The Shapiro–Wilk test assessed the normality of the study variables. The Box–Cox transformation was applied to variables that did not satisfy the normality assumption. Additionally, variables with repeated measurements over time were evaluated by adjusting the data to correlation structures based on the AIC (Akaike Information Criterion) [90]. Additionally, the residuals, kurtosis, and homogeneity of variance were visually inspected after the transformations to verify the proper fulfillment of statistical assumptions [91]. A mixed linear model was used to perform the analysis of variance (ANOVA). Also, Tukey’s multiple comparison tests (*p* ≤ 0.05) helped to determine differences between treatments. Data associated with levels of macro- and micronutrients were adjusted using a second-degree polynomial regression model, obtaining the 95% confidence intervals, R^2^, R^2Adj^, and the *p*-value from the F test (*p* ≤ 0.05). Finally, principal component analysis (PCA) facilitated the exploration of the linear relationship among the study variables in both groups of plants.

## 5. Conclusions

*C*Las spreads more slowly in *C. latifolia* compared to susceptible citrus varieties, leading to a more gradual expression of symptoms. The disease affected plant development and physiological parameters related to photosynthesis, altering vascular structures and increasing starch accumulation in the leaf’s midribs and mesophyll. Additionally, an increase in the endogenous content of salicylic acid and its biosynthesis precursors is confirmed in diseased plants compared to healthy ones. However, it was observed that the levels of benzoic acid in the leaves were higher, while the levels of salicylic acid were lower than in findings reported from susceptible citrus varieties. This finding may suggest a reduced conversion of benzoic acid to salicylic acid. Regarding the impact of combinatorial treatments using macro- and micronutrients, it was concluded that soil application of low doses of macronutrients in combination with micronutrients (Ma-1 + Mi-2) resulted in less damage to Persian lime plants affected by HLB. This treatment promoted more significant plant development, improved photosynthetic performance, and reduced metabolic alterations, such as starch accumulation in the leaves of diseased plants. Applying the fertilizer consistently and at lower doses may improve its utilization. In contrast, increasing the doses of macronutrients and simultaneously applying micronutrients to the soil and through foliar sprays does not seem advantageous, even for healthy Persian lime plants, under the conditions evaluated. Thus, careful fertilization management may optimize its use and help reduce the negative effects of HLB on diseased Persian lime plants. It is essential to reassess the timing and dosage of fertilization practices in regions where HLB has become prevalent, such as in the citrus-growing states of Mexico. This reevaluation could lead to potential benefits in environmental sustainability and production costs.

## Data Availability

Data is contained within the article or Supplementary Material.

## Supplementary Materials

The following supporting information can be downloaded here: https://www.mdpi.com/article/10.3390/plants14071086/s1. Figure S1: Leaf reflectance percentage at (A) 550 nm and (B) 790 nm for macro- and micronutrient levels. According to Tukey (*p* < 0.05), different letters represent significant differences. Values represent four replicates, mean ± standard deviation. Ma = macronutrients, Mi = micronutrients, ns= not significant. Figure S2: Effect of *C*Las infection and fertilization treatments on the content of (A) total chlorophyll, (B) sucrose, and (C) starch in leaves. The main effects, according to the general linear model (ANOVA), are indicated in graphs. According to Tukey (*p* < 0.05), different letters represent significant differences. The values represent four replicates, mean ± standard deviation. Ma = macronutrients, Mi = micronutrients. Significant *p*-values are shown in bold. Figure S3: Histological sections of leaf midribs show accumulation of starch grains in collenchyma, parenchyma, and pith cells in HLB-diseased plants vs. healthy plants. Diseased plants show anatomical changes in the vascular system, such as hyperplasia and increased phloem area. coll = collenchyma, par = parenchyma, Pf = phloem fibers, P = phloem, X = xylem, pi = pith, H = hyperplasia. Black arrows indicate the presence of starch. Figure S4: Effect of *C*Las infection and fertilization treatments on cumulative growth in (A) height, (B) canopy volume, and (C) trunk diameter. According to the general linear model (ANOVA), graphs indicate the main effects. According to Tukey (*p* < 0.05), different letters represent significant differences. Values represent four replicates, mean ± standard deviation. Ma = macronutrients, Mi = micronutrients. Significant *p*-values are shown in bold. Figure S5: Multivariate principal component analysis (biplot-PCA), including the study variables and their association with the groups of healthy and HLB-diseased Persian lime plants. Ht = height, CV = canopy volume, DT = trunk diameter, Chl = chlorophyll, Suc = sucrose, Glc = glucose, St = starch, tCA = *trans*-cinnamic acid, BA = benzoic acid, SA = salicylic acid, Refl = reflectance, Fv/Fm = photosystem II efficiency. Table S1: Fertilization treatments evaluated in healthy and HLB-diseased plants, consisting of three levels of macro- and micronutrients. Table S2: Validation of a chromatographic method for the quantification of BA, *t*-CA, and SA.
