# Supplementary material for: Fertilization Strategies in Huanglongbing-Infected Citrus latifolia and Their Physiological and Hormonal Effects
Source: Plants (Basel). 2025 Apr 1;14(7):1086. doi: 10.3390/plants14071086 (PMC11991268; doi:10.3390/plants14071086)
Supplement: Supplementary file 1 [file plants-14-01086-s001.zip › plants-3481995-supplementary.pdf]

Fertilization Strategies in Huanglongbing-Infected *Citrus latifolia* and Their Physiological and Hormonal Effects

Luis A. Pérez-Zarate [1], Aída Martínez-Hernández [2], Francisco Osorio-Acosta [1], Eliseo García-Pérez [1], Fredy Morales-Trejo [1] and Juan A. Villanueva-Jiménez [1,*]

[1] Colegio de Postgraduados, Campus Veracruz, Km. 88.5 Carretera Fed. Xalapa-Veracruz, Manlio F. Altamirano 91690, Veracruz, Mexico; alfredo.perez@colpos.mx (L.A.P.-Z.); fosorioa@colpos.mx (F.O.-A.); geli-seo@colpos.mx (E.G.-P); fredymt@hotmail.com (F.M.-T).

[2] Colegio de Postgraduados, Campus Campeche, Carretera Haltún-Edzná km 17.5, Sihochac, Champotón 24450, Campeche, Mexico; aida.martinez@colpos.mx (A.M.-H).

* Correspondence: javj@colpos.mx (J.A.V.-J.); Tel.: +52-2292-010770 Ext. 3037.

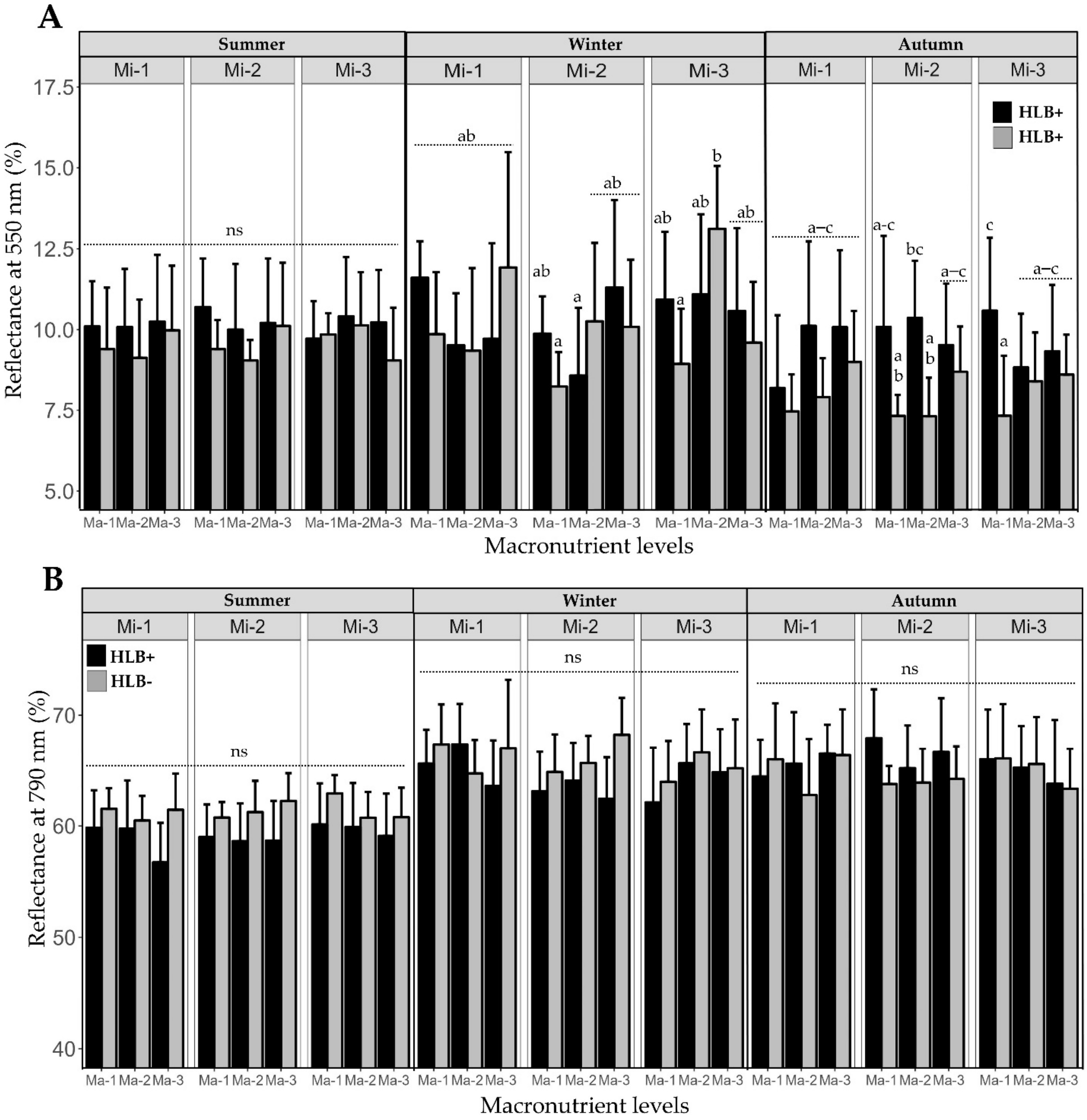

Figure S1: Leaf reflectance percentage at A) 550 nm and B) 790 nm for macro- and micronutrient levels. According to Tukey ($p < 0.05$), different letters represent significant differences. Values represent four replicates, mean ± standard deviation. Ma = macronutrients, Mi = micronutrients, ns= not significant.

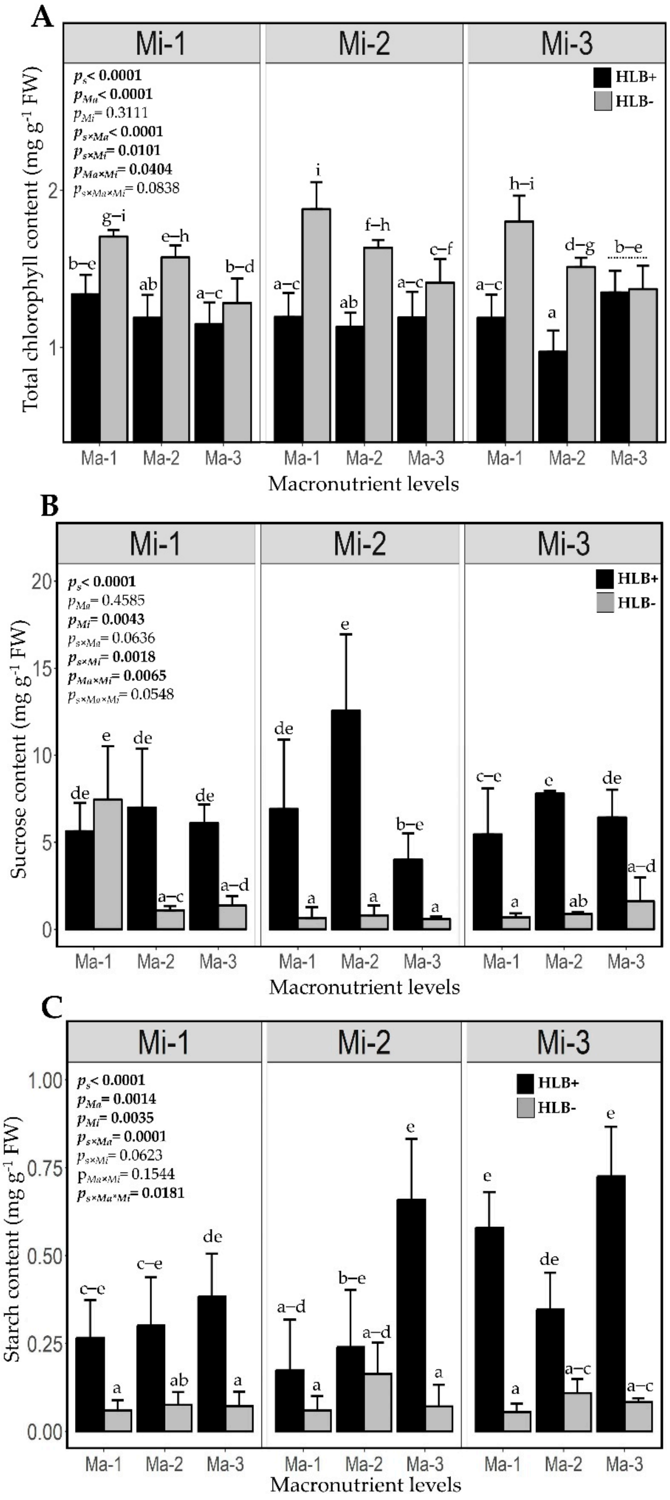

Figure S2: Effect of *C*Las infection and fertilization treatments on the content of A) total chlorophyll, B) sucrose, and C) starch in leaves. The main effects, according to the general linear model (ANOVA), are indicated in graphs. According to Tukey ($p < 0.05$), different letters represent significant differences. The values represent four replicates, mean ± standard deviation. Ma = macronutrients, Mi = micronutrients. Significant *p*-values are shown in bold.

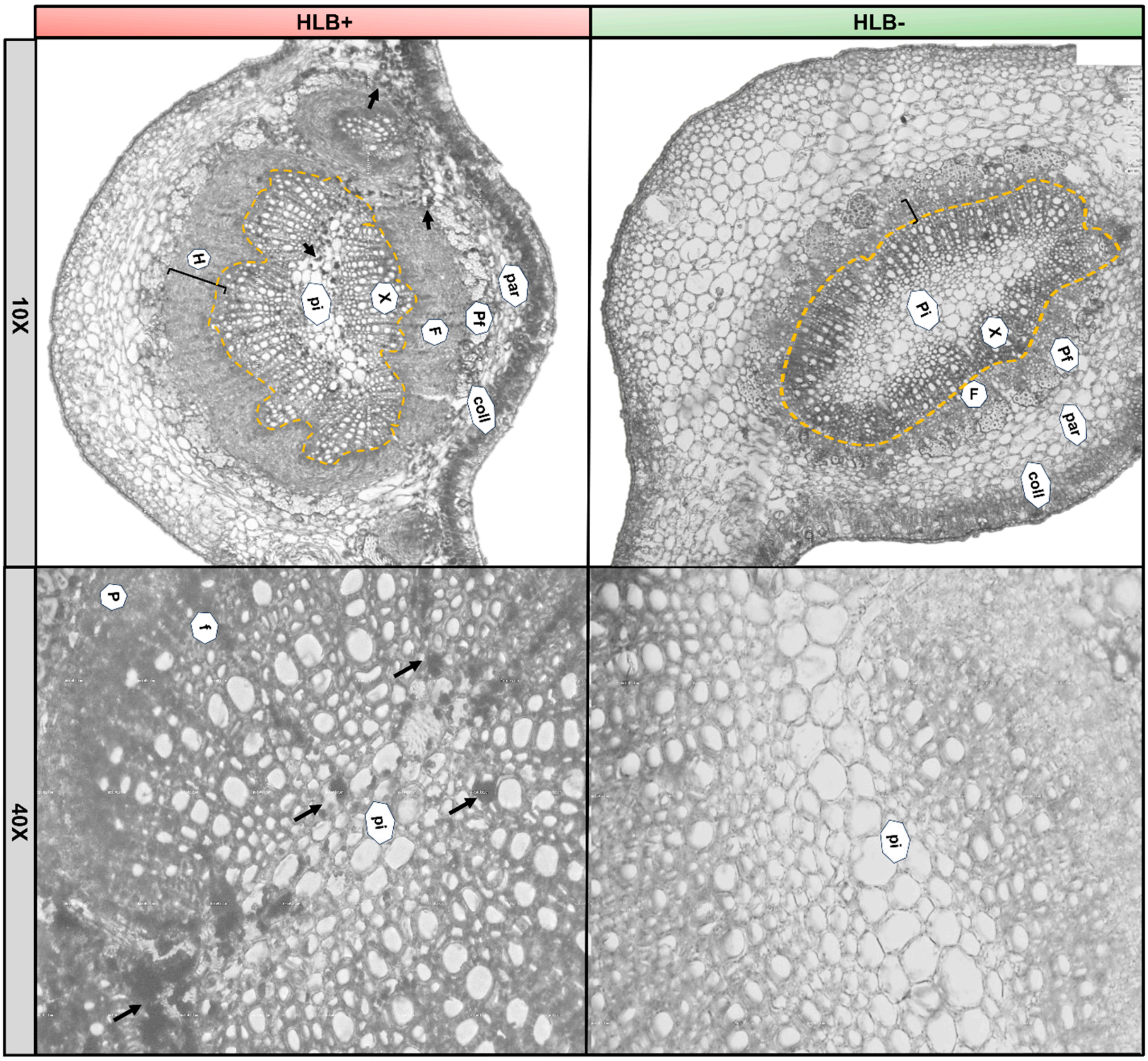

Figure S3: Histological sections of leaf midribs show accumulation of starch grains in collenchyma, parenchyma, and pith cells in HLB-diseased plants, vs. healthy plants. Diseased plants show anatomical changes in the vascular system, such as hyperplasia and increased phloem area. coll = collenchyma, par = parenchyma, Pf = phloem fibers, P = phloem, X = xylem, pi = pith, H = hyperplasia. Black arrows indicate the presence of starch.

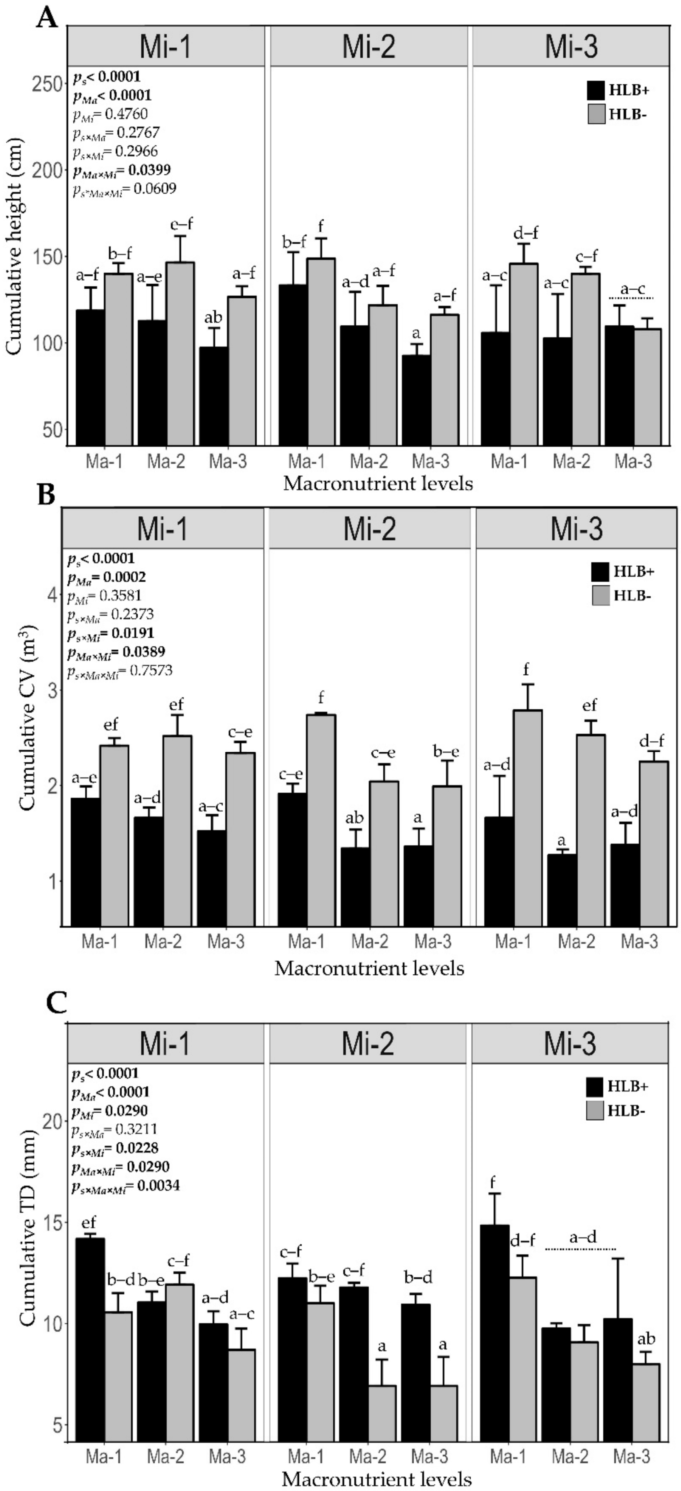

Figure S4: Effect of *C*Las infection and fertilization treatments on cumulative growth in A) height, B) canopy volume, and C) trunk diameter. According to the general linear model (ANOVA), graphs indicate the main effects. According to Tukey ($p < 0.05$), different letters represent significant differences. Values represent four replicates, mean ± standard deviation. Ma = macronutrients, Mi = micronutrients. Significant *p*-values are shown in bold.

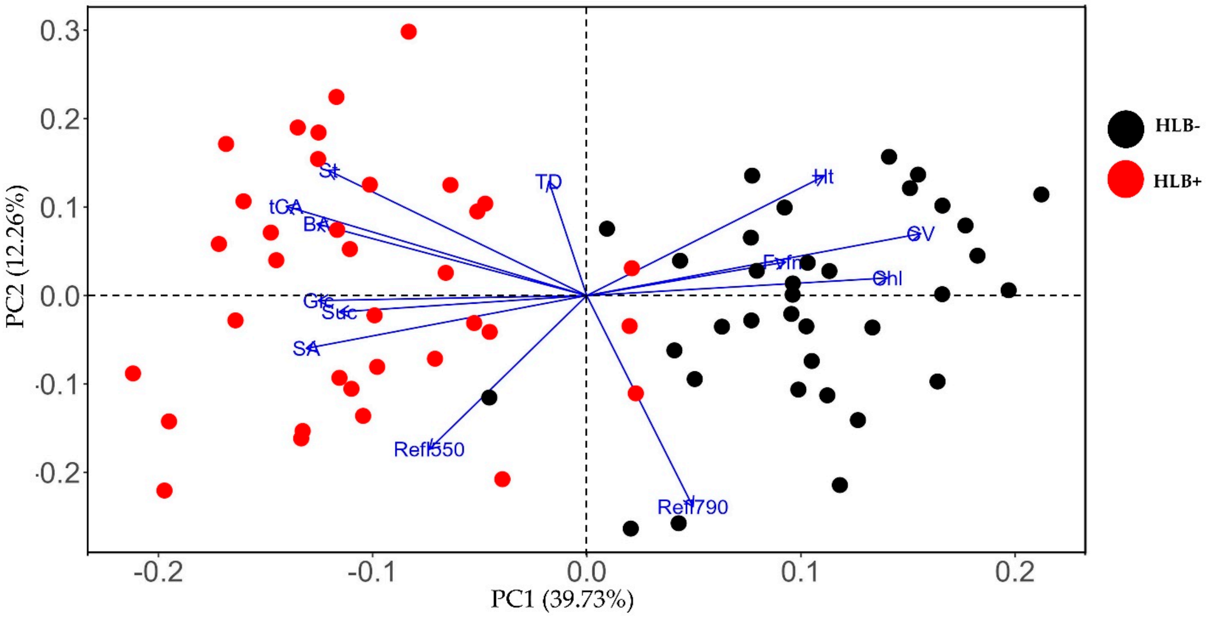

Figure S5: Multivariate principal component analysis (biplot-PCA), including the study variables and their association with the groups of healthy and HLB-diseased Persian lime plants. Ht = height, CV = canopy volume, DT = trunk diameter, Chl = chlorophyll, Suc = sucrose, Glc = glucose, St = starch, tCA = *trans*-cinnamic acid, BA = benzoic acid, SA = salicylic acid, Refl = reflectance, Fv/Fm = photosystem II efficiency.

Table S1: Fertilization treatments evaluated in healthy and HLB-diseased plants, consisting of three levels of macro- and micronutrients.

Levels		Dose (g plant^{-1} per application^{-1}) to the soil										Foliar dose per application
Macros	Micros	N	P	K	Ca	Mg	S	Zn	B	Mn	Fe	
Ma-1	Mi-1	3	0.5	0.8								0.9 g L^{-1} (1.1%B, 1.3% Zn, 6% Fe, 2.4% Mn, 0.25% Cu, 0.25% Mo)¶
	Mi-2	3	0.49	0.81	0.37	0.38	0.36	0.001	0	0.001	0.009	
	Mi-3	3	0.49	0.81	0.37	0.38	0.36	0.001	0	0.001	0.009	
Ma-2	Mi-1	6	1	1.6	1.11	0.74						4 mL L^{-1} (4.8% N, 4.9% Mg, 4.9%B, 9.9% Zn)Π
	Mi-2	6.00	1.13	1.60	1.30	1.22	0.68	0.30	0.15	0.30	0.01	
	Mi-3	6.00	1.13	1.60	1.30	1.22	0.68	0.30	0.15	0.30	0.01	
Ma-3	Mi-1	7.00	1.60	2.00	1.20	0.80						4 mL L^{-1} (4.8% N, 4.9% Mg, 4.9%B, 9.9% Zn)Π
	Mi-2	7.00	1.60	2.00	1.28	1.23	0.70	0.30	0.15	0.30	0.01	
	Mi-3	7.00	1.60	2.00	1.28	1.23	0.70	0.30	0.15	0.30	0.01	

¶Ultrasol Micro®, ΠMagzibor.

Table S2: Validation of a chromatographic method for the quantification of BA, *t*-CA, and SA.

Parameter[a]	*Trans*-cinnamic acid	Benzoic acid	Salicylic acid
Linear range (ng μL^{-1})	0.2-10	1-100	0.2-10
Linear regression equation	y = 0.048x - 0.0081	y = 0.0463x + 0.0661	y = 0.0278x - 0.0032
R^2	0.9997	0.9985	0.9998
Precision[b] (%RSD)	11.18	5.14	7.4
Recovery (%)	97.73 ± 8.12	80.74 ± 6.55	104.73 ± 8.87
Limit of detection (ng g^{-1})	2.84	52.72	8.72
Limit of quantification (ng g^{-1})	8.61	159.75	26.42
Relative retention time (% RSD)	0.0095	0.0549	0.0237

[a]n = 6.
